# Development and Internal Validation of an Explainable Machine Learning Model for Predicting Buttock Claudication After EVAR: A Dual-Center Cohort Study

**DOI:** 10.3390/bioengineering13060665

**Published:** 2026-06-08

**Authors:** Yajing Li, Hongru Deng, Yongquan Gu

**Affiliations:** 1 Department of Vascular Surgery, Xuanwu Hospital, Capital Medical University, Beijing 100053, China; 2 Department of Vascular Surgery, Fu Xing Hospital, Capital Medical University (FXH-CMU), Beijing 100038, China

**Keywords:** endovascular aneurysm repair (EVAR), buttock claudication, internal iliac artery embolization, explainable machine learning, risk prediction model

## Abstract

Buttock claudication after endovascular aneurysm repair (EVAR) impairs recovery and quality of life, yet individualized preoperative risk tools are scarce. We conducted a retrospective dual-center cohort study of consecutive EVAR patients from Fuxing and Xuanwu Hospitals. The endpoint was new-onset postoperative buttock claudication. Missingness was quantified for each predictor and handled using complete-case analysis or model-based single imputation according to the extent of missingness. Data were split into training and held-out test sets at a 70:30 ratio with outcome stratification. Predictor screening, preprocessing, and hyperparameter tuning were performed within the training/resampling framework to minimize data leakage. Ten algorithms were tuned using stratified 10-fold cross-validation, and test set performance was assessed using discrimination, threshold-based metrics, calibration plots, calibration intercept/slope, Brier score, and decision-curve analysis. SHapley Additive exPlanations (SHAP) provided model-agnostic explanations. A web calculator was deployed. Among 272 patients, 71 (26.1%) developed claudication. Independent risk factors included aneurysm with iliac involvement (adjusted OR 4.04), male sex (3.26), unilateral (3.86) and bilateral internal iliac artery embolization (8.61), and hyperlipidemia (5.66); >2 distal internal iliac branches was protective (0.15). On the test set, the neural network achieved the highest AUROC (test ROC), with the highest sensitivity (0.810) and top F1 (0.557) at balanced specificity (0.617); CatBoost maximized accuracy (0.790) and specificity (0.900). Calibration was acceptable, and DCA showed positive net benefit across clinically plausible thresholds. SHAP confirmed physiologic directions and enabled case-level interpretation. An explainable machine learning framework accurately stratifies risk of buttock claudication after EVAR, highlighting the roles of internal iliac embolization, iliac involvement, and distal branch anatomy. The publicly available Shiny tool supports perfusion-aware planning and shared decision-making.

## 1. Introduction

Endovascular aneurysm repair (EVAR) has become the default approach for infrarenal abdominal and iliac aneurysms; however, pelvic ischemic symptoms—most notably buttock claudication—remain a meaningful complication [1,2]. These symptoms can reduce walking capacity, delay rehabilitation, and impair postoperative quality of life. Strategies used to achieve an adequate seal and appropriate limb alignment (intentional internal iliac artery [IIA] embolization being one prime example) may reduce pelvic inflow, whereas the adequacy of collateral circulation varies considerably across patients and is difficult to assess prospectively. Routine practice in making decisions regarding preservation of one or both IIAs, staging of such procedures, selective embolization, or even small adjustments to landing zones, relies heavily on anatomical feasibility and clinician judgment [3,4]. The incidence of buttock claudication itself is non-trivial; patient specific vulnerability combines both anatomic factors and various forms of systemic vascular burden. A robust preoperative risk stratification tool is therefore required; planning, IIA-preservation strategies, patient counseling, and all that follows from it, must be at least partially guided by such a tool.

Buttock claudication after EVAR is fundamentally a problem of pelvic hypoperfusion. The IIA supplies the superior/inferior gluteal and obturator systems; intentionally embolizing or covering one or both IIAs to secure an endograft seal forces the gluteal bed to rely on various collateral routes [5,6,7,8]. Under exertion these circuits can become insufficient, creating the well-described gradient of risk: no IIA interruption → unilateral → bilateral loss of inflow. Anatomic complexity further modulates this risk; iliac-involved aneurysms specifically often require longer landing zones, adjunctive embolization, or limb extensions that directly encroach upon pelvic inflow. The actual capacity for collateralization likely depends on distal IIA arborization—number and/or caliber of those branches representing some form of native reserve. Systemic vascular milieu also plays a part: peripheral arterial disease, dyslipidemia and all associated cumulative exposures impair both macro and microvascular function, decreasing all aspects of vasodilatory reserve; sex-related differences in muscle mass and subsequent oxygen demand during ambulation are another potential contributor [9,10]. General attributes like age, BMI, or simple aortic diameter show inconsistent associations when pelvic inflow/outflow is taken into account. Collectively, anatomic and clinical determinants of risk interact in near-constant, individualized ways. Risk stratification should and must be, to some degree, mechanism-based.

Despite decades of EVAR experience, most studies on buttock claudication remain single-center, small, and heterogeneous in design [11,12]. Variable outcome definitions and follow-up, limited adjustment for confounding, the near absence of calibration and formal clinical-utility assessment remain common limitations. Many models emphasize AUROC alone; post hoc threshold selection, risk train–test leakage, and class imbalance are commonly unaddressed problems. Anatomic details that likely matter—the number of distal internal iliac branches being one example, structured collateral scores another—are often missing or inconsistently coded; systemic vascular burden is coarsely captured. Where machine learning has been attempted, black-box models are the norm; transparent, patient-level explanations are uncommon, external validation and all subsequent ‘deployable’ tools are rare, and integration into clinical workflows has not been demonstrated. Clinicians still lack a rigorous, interpretable and usable preoperative risk tool to personalize IIA preservation, selective/staged embolization, and to some extent postoperative planning.

Risk for buttock claudication emerges from nonlinear interactions among anatomic (embolization status, iliac involvement, branch count) and clinical factors. Traditional regression captures average effects but misses higher-order patterns and all associated thresholds (bilateral vs. single, for instance, and their synergistic effects). A multi-algorithm ML strategy is therefore appropriate to model these complex boundaries; prespecified safeguards are required—screening (*p* < 0.05), at least a 70/30 train-test split, some form of cross-validation, locked thresholds, and comprehensive evaluation (AUROC, AUPRC, calibration, decision curves). Avoiding the ‘black-box’ adoption barriers, we embed model-agnostic explainability via SHapley Additive exPlanations (SHAP) to support all of the above-mentioned clinical decisions. Individualized and, to the end-user at least, partially transparent, risk stratification is a realizable goal in EVAR.

We aimed to build an individualized, clinically usable risk model for postoperative buttock claudication following EVAR using a dual-center cohort. Specifically, we prespecified feature screening within the training set at *p* < 0.05, then developed and benchmarked ten supervised algorithms under a 70/30 train–test split with stratified cross-validation and locked thresholds prior to formal test evaluation. Comprehensive performance was reported—AUROC, accuracy, various forms of sensitivity/specificity, precision, F1, calibration and decision-curve analysis; confusion matrices were used to visualize more subtle error structures. Global variable importance across models and both global and case-level SHAP explanations for the ‘top performing’ test set model were provided to ensure at least some degree of physiological coherence and patient-level transparency [13]. The present study was designed to develop and internally validate an explainable preoperative prediction model for postoperative buttock claudication after EVAR. Its main contribution is the integration of clinically relevant pelvic perfusion-related variables, including internal iliac artery embolization status, iliac artery involvement, and distal internal iliac branch anatomy, into a transparent risk-prediction workflow. The model was benchmarked against logistic regression and other machine learning algorithms, interpreted using SHAP, and implemented as a research-use Shiny prototype for individualized risk estimation.

## 2. Materials and Methods

### 2.1. Cohort and Baseline Characteristics

This retrospective, dual-center cohort study included consecutive adults who underwent EVAR at Fuxing Hospital, Capital Medical University, and Xuanwu Hospital, Capital Medical University. The time frame of inclusion was from 1 January 2017 to 1 July 2025. The protocol was approved by the Ethics Committee of Fuxing Hospital (approval No. 2025FXHEC-KSP054); waiver of consent was obtained. Prespecified eligibility criteria were used: inclusion required age ≥ 18 years, some form of index EVAR for infrarenal abdominal aortic aneurysm (AAA), iliac involvement of the AAA, or isolated iliac artery aneurysm; baseline perioperative data and subsequent follow-up sufficient to ascertain the desired endpoint were also required. Exclusions were open aortic repair, any thoracic/thoracoabdominal endovascular procedure without an EVAR component; prior EVAR (only the first was analyzed), repeat/revision EVAR during the study window; documented pre-existing buttock claudication or other clearly confounding alternative conditions (severe degenerative hip/knee disease being a prime example, neurogenic being another); perioperative death prior to outcome ascertainment; or missing primary outcome data or unavailable key anatomical or procedural predictor information required for model development. Missingness was assessed for each candidate predictor before model development. No missing values were observed in the variables used for final model development; therefore, imputation was not performed in the final analysis. Candidate predictors were extracted from electronic records and operative notes: Site_of_aneurysm, Gender, Peripheral_arterial_disease, Number_of_internal_iliac_arteries_embolized, Chronic_obstructive_pulmonary_disease, Chronic_kidney_disease, Antiplatelet, Hyperlipidemia, History_of_previous_abdominal_and_pelvic_surgery, Cardiovascular_disease, Marital_status, Smoking, Drinking, Cerebrovascular_disease, Hypertension, Diabetes, Number_of_distal_internal_iliac_artery_branches, Age, BMI, and Maximum_diameter_of_abdominal_aorta. The primary endpoint was new-onset buttock claudication after EVAR—exertional gluteal pain relieved by rest—adjudicated from standardized ward/clinic documentation within a prespecified postoperative window. Categorical variables were ordinal-encoded, and continuous variables were inspected for distributional characteristics and outliers. Missingness was first quantified for each candidate predictor before model development. Variables with low-level missingness were handled using complete-case analysis for the corresponding analyses, whereas variables with non-negligible missingness were handled using model-based single imputation based only on information available within the training data. To avoid information leakage, imputation, scaling, encoding, and any other preprocessing steps were incorporated into the resampling pipeline and were not estimated using the held-out test set.

### 2.2. Feature Screening in the Training Set

After eligibility, the data were randomly split into training (70%) and test (30%) sets; stratification by the endpoint and a fixed random seed was used to ensure adequate representation. Feature screening was performed exclusively within the training set. Univariable logistic regressions were fitted for each candidate predictor; variables meeting the prespecified screening threshold (*p* < 0.05) were entered into a subsequent multivariable model. A full multivariable logistic regression was then fitted, and multicollinearity was assessed using variance inflation factors(VIF < 5 being acceptable), and all adjusted odds ratios (ORs) with their corresponding 95% confidence intervals were reported. The specific variables that remained in the final model comprised the feature set for any downstream machine learning developments.

### 2.3. Model Development and Internal Validation (Training Set)

Ten supervised algorithms were trained on the multivariable-selected features: AdaBoost, CatBoost, K-nearest neighbors (KNNs), LightGBM, Logistic regression, neural network, Random Forest, Support Vector Machine (SVM), XGBoost, and Gradient Boosting Machine (GBM). Pipelines were tailored per model (e.g., standardization for KNN/SVM/neural network; no scaling for tree/boosting models), with class weighting where applicable. Hyperparameters were tuned using stratified 10-fold cross-validation within the training set, with the AUROC as the primary optimization metric and the AUPRC and F1 score used as secondary considerations. Calibration was evaluated using calibration plots, calibration intercept, calibration slope, and the Brier score. If substantial miscalibration was observed during internal validation, probability recalibration was performed within the same cross-validation framework. Classification thresholds were selected using training-set predictions, including Youden’s J statistic and F1-maximizing thresholds, and were locked before application to the held-out test set. Feature selection, preprocessing, hyperparameter tuning, calibration, and threshold selection were performed exclusively within the training/resampling framework, and the held-out test set was not used for any model fitting, preprocessing, tuning, or threshold selection.

### 2.4. Independent Test Set Evaluation

All tuned models were frozen and applied to the independent 30% test set without any further fitting or threshold adjustment. The same discrimination, calibration, and classification metrics were computed; ROC, calibration, and DCA plots were generated accordingly. Confusion matrices were created to visualize more specific false-positive/false-negative patterns relative to these locked thresholds. Quantifying uncertainty in the discrimination aspect was of particular interest; bootstrap 95% CIs for AUROC were obtained (1000 resamples, stratified by outcome). The algorithm achieving the highest test set AUROC—with both F1 and various forms of calibration as secondary considerations—was designated the ‘primary’ model; all subsequent interpretation and potential deployment followed from this choice.

### 2.5. Explainability and Clinical Deployment

We summarized global variable importance for each algorithm using model-appropriate approaches. The best-performing model on the test set—the neural network—was given more specific attention; SHAP values were computed to provide both global and local interpretability. Outputs included a global bar plot and associated beeswarm distribution to rank/illustrate feature contributions, various forms of dependence plots for key predictors, and more representative force and waterfall plots to decompose at least some individual predictions. The locked primary model was then implemented in a bedside Shiny web application: study predictors are entered as inputs, individualized predicted risk follows, along with an explained panel, all to some degree rooted in the previous SHAP work. All analyses were performed in R 4.5.0; version-controlled scripts were used throughout to maintain all aspects of reproducibility.

## 3. Results

### 3.1. Cohort and Baseline Characteristics

We included 272 EVAR patients; 71 (26.1%) developed postoperative buttock claudication and the remaining 201 did not. Variables are summarized in Table 1. Patients with buttock claudication more often had an abdominal aortic aneurysm with associated iliac artery aneurysm (47.9% vs. 26.4%; *p* = 0.001) and some form of peripheral arterial disease (54.9% vs. 34.8%; *p* = 0.005). Internal iliac artery embolization, whether unilateral or bilateral, was more frequent among patients with claudication (overall *p* < 0.001). Hyperlipidemia was also significantly more prevalent (87.3% vs. 66.2%), while a history of previous abdominal/pelvic surgery was less common (29.6% vs. 44.3%). A reduced number of distal internal iliac artery branches (≤2) was markedly enriched in the cases. Age (65.0 ± 8.3 vs. 65.7 ± 7.6 years), BMI (29.8 ± 4.5 kg/m^2^) and maximum abdominal aortic diameter (5.7 ± 0.5 cm) showed no significant difference between the two groups; all other comorbidities and near-identical lifestyle factors were balanced. The cohort was then randomly split 70%/30% into training and test sets to allow for both model development and subsequent independent evaluation.

### 3.2. Feature Screening in the Training Set

In the training set (n = 191; buttock claudication 50/191, 26.2%), both univariable and multivariable logistic regression identified several independent risk factors for postoperative buttock claudication (Table 2). On multivariable analysis, abdominal aortic aneurysm with associated iliac artery aneurysm (adjusted OR 4.04, 95% CI 1.73–9.40, *p* = 0.001), male sex (adjusted OR 3.26, 95% CI 1.20–8.86, *p* = 0.021), unilateral internal iliac artery embolization (adjusted OR 3.86, 95% CI 1.38–10.80, *p* = 0.010), bilateral internal iliac artery embolization (adjusted OR 8.61, 95% CI 1.78–41.68, *p* = 0.007), and hyperlipidemia (adjusted OR 5.66, 95% CI 1.84–17.37, *p* = 0.002) were independently associated with higher odds of postoperative buttock claudication, whereas having >2 distal internal iliac artery branches was the only independent protective factor (adjusted OR 0.15, 95% CI 0.05–0.41, *p* < 0.001). Other comorbidities and all continuous variables (age, BMI, and maximum abdominal aortic diameter) were not independently associated after adjustment. Variables retained in the final multivariable model were then used as inputs for subsequent machine learning development.

### 3.3. Model Development and Internal Validation (Training Set)

Using the multivariable-selected predictors, we trained ten algorithms on the 70% training split and reported performance at model-specific probability thresholds (Table 3). Operating points showed complementary trade-offs: LightGBM achieved the highest F1 (0.656), with a near-perfect balance of sensitivity and specificity; KNN and SVM followed closely thereafter. Random Forest yielded the highest accuracy (0.822), driven by extremely high specificity but low sensitivity, all other metrics being compromised; GBM prioritized some form of recall (sensitivity was ~0.88) at the expense of the previously mentioned specificity. Logistic regression and the neural network produced identical summaries at their chosen thresholds. Full thresholds and point estimates for every model are provided in Table 3.

**Figure 1:**
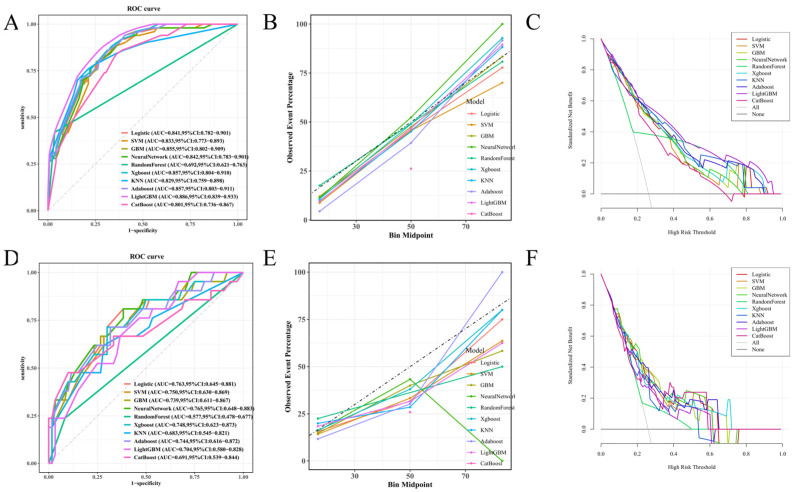
Discrimination, calibration, and clinical utility of the models. (**A**–**C**) Training/internal-validation set: (**A**) ROC curves with AUROC for each algorithm; (**B**) calibration plots comparing predicted versus observed risk (smoothed curve and ideal 45° line); (**C**) decision-curve analysis (DCA) showing net benefit across threshold probabilities. (**D**–**F**) Held-out test set: (**D**) ROC curves; (**E**) calibration plots; (**F**) DCA. Thresholds used in DCA and confusion matrices were locked from the training phase. Higher curves indicate better performance. Abbreviations: AUROC, area under the ROC curve; DCA, decision-curve analysis.

**Figure 2:**
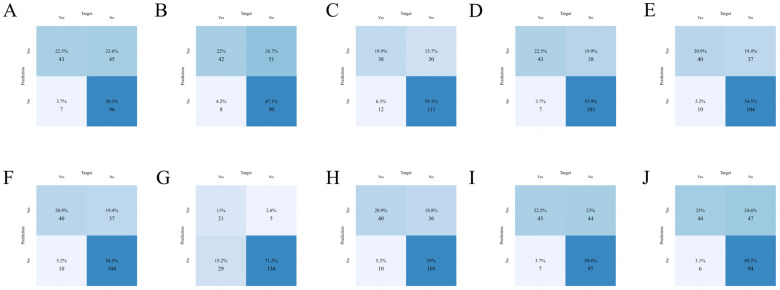
Confusion matrices for the training/internal validation set (positive class = postoperative buttock claudication). Matrices depict true/false positives and true/false negatives at the locked operating threshold for each algorithm, ordered as: (**A**) AdaBoost, (**B**) CatBoost, (**C**) KNN, (**D**) LightGBM, (**E**) logistic regression, (**F**) neural network, (**G**) Random Forest, (**H**) SVM, (**I**) XGBoost, (**J**) GBM. Darker cells indicate higher counts. These visualizations highlight models that favor sensitivity versus specificity and reveal dominant error modes, and the blue color gradient reflects cell frequency, with darker blue indicating a higher count/proportion.

Discrimination, calibration, and to a large extent clinical utility on the training data are visualized in Figure 1A–C (ROC, calibration, DCA); qualitatively these mirror the trade-offs previously described. Error profiles are shown as confusion matrices for each method (Figure 2, ordered by model): AdaBoost, CatBoost, KNN, LightGBM, Logistic, neural network, Random Forest, SVM, XGBoost, GBM. Focusing on the more ‘pure’ specificity or sensitivity favored models, and all intermediate cases, the various models show a range of clinically useful behaviors.

### 3.4. Independent Test Set Evaluation

**Figure 3:**
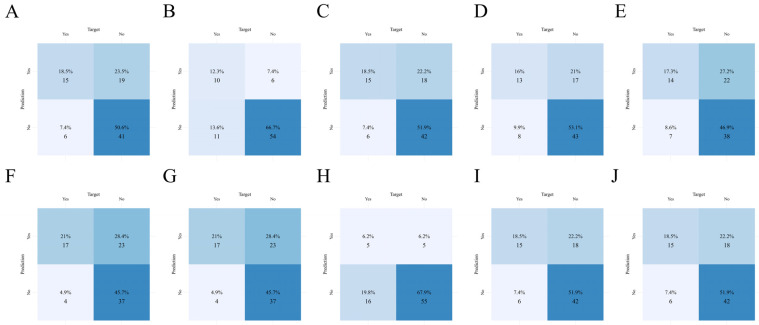
Confusion matrices for the held-out test set (positive class = postoperative buttock claudication). (**A**) AdaBoost, (**B**) CatBoost, (**C**) KNN, (**D**) LightGBM, (**E**) logistic regression, (**F**) neural network, (**G**) Random Forest, (**H**) SVM, (**I**) XGBoost, (**J**) GBM. Thresholds are identical to those fixed on the training data. Patterns illustrate generalizability of each model’s error profile, and the blue color gradient reflects cell frequency, with darker blue indicating a higher count/proportion.

On the held-out test set, overall performance varied across algorithms at their model-specific operating thresholds (Table 4). Accuracy spanned from 0.642 to 0.790; CatBoost achieved the highest accuracy (the latter being accompanied by near-perfect specificity, 0.900) though a more moderate F1 of 0.541. Random Forest maximized some form of specificity (0.917) and precision, sensitivity and consequently F1 being severely compromised (0.238/0.323). Logistic regression and the neural network delivered the highest sensitivity (both at 0.810) with top tier F1 scores; balanced specificity was also obtained for them (approximately 0.617). SVM, GBM, and to a large extent XGBoost showed very similar balanced profiles (accuracy, sensitivity and F1 all around 0.704–0.714), KNN being the outlier with lower F1. Discrimination, calibration and various clinical utility curves for the test set are detailed in Figure 1D–F (ROC, calibration, DCA); the neural network was selected as the primary model for downstream explainability based on these metrics. Per-model error patterns are visualized through the test set confusion matrices (Figure 3, order as above), where specificity-oriented models (Random Forest being a prime example) have, and should have, fewer false positives; sensitivity or ‘all round’ models are of secondary clinical importance.

### 3.5. Explainability and Clinical Deployment

**Figure 4:**
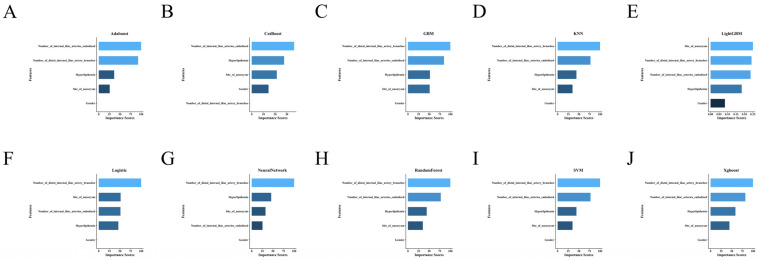
Global variable importance across algorithms. Importance summarized on a comparable scale. IIA embolization status, distal IIA branch count, and iliac-involved aneurysm consistently rank highest. (**A**) AdaBoost; (**B**) CatBoost; (**C**) GBM; (**D**) KNN; (**E**) LightGBM; (**F**) logistic regression; (**G**) neural network; (**H**) Random Forest; (**I**) SVM; (**J**) XGBoost.

**Figure 5:**
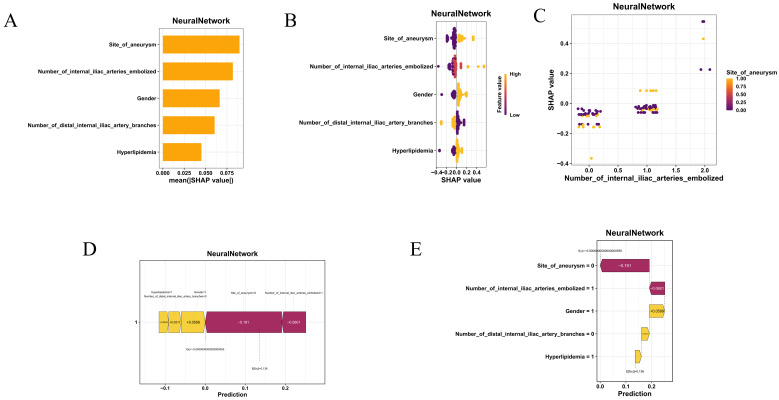
SHAP explainability for the best-performing model (neural network). (**A**) Global SHAP bar plot ranking features by mean absolute SHAP value (overall contribution). (**B**) Beeswarm plot: each point represents a patient; horizontal position indicates SHAP value (impact on risk), and color encodes feature value (low to high). (**C**) SHAP dependence plots for key predictors (e.g., IIA embolization status, distal branch count, iliac involvement), optionally with interaction hints. (**D**) Representative SHAP force plot decomposing an individual prediction into prediction-increasing positive contributions (yellow) and prediction-decreasing negative contributions (purple). (**E**) Waterfall plot illustrating cumulative SHAP contributions from baseline risk to the final predicted probability. Positive SHAP values raise, and negative values lower, the predicted risk.

**Figure 6:**
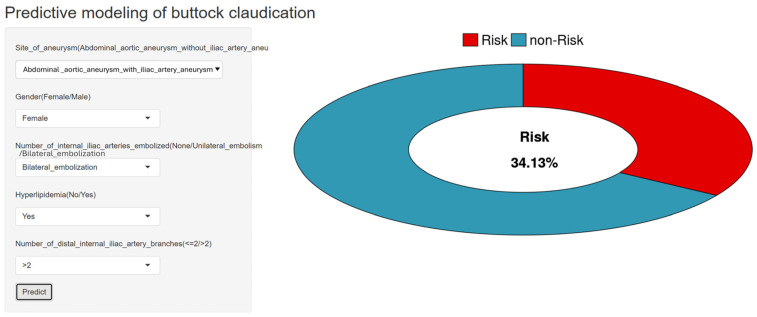
Web-based clinical calculator for individualized risk estimation. Screenshot of the deployed Shiny application showing input fields for study predictors and the resulting predicted probability of postoperative buttock claudication with an accompanying explanation panel aligned to SHAP outputs. The tool is accessible at https://cacsriskmodel.shinyapps.io/makee/, accessed on 7 October 2025.

Across various algorithms, the global variable importance profiles (Figure 4) consistently identified internal iliac artery embolization status (bilateral/unilateral), the number of distal internal iliac branches, and aneurysm site (specifically abdominal aortic with some form of iliac involvement) as the most influential predictors. Contributions were directionally concordant with multivariable logistic results: embolization and associated iliac involvement being risk-increasing, and having >2 distal branches risk-decreasing. Focusing on the top-performing neural network, SHAP analyses (Figure 5A–E) provided both global and near individual-level transparency; the bar plot and subsequent beeswarm confirmed the features mentioned above as dominant drivers; dependence plots illustrated stepwise (categorical) risk shifts—none → unilateral → bilateral being a prime example—and the previously noted monotonic decrease with increasing branch count; representative force/waterfall plots decomposed specific patient predictions, embolization, iliac factors, male sex and to a large extent hyperlipidemia pushing the prediction ‘up’ while all or part of the distal branch effect pulls it down. These effect directions were and remain clinically plausible. A bedside usable interactive web calculator was developed; Figure 6 shows the interface, currently available at https://cacsriskmodel.shinyapps.io/makee/, accessed on 7 October 2025. Individual risk estimates can be generated from any set of patient features, with all or part of the model explanation provided to the clinician.

## 4. Discussion

In this dual-center retrospective cohort study (2017–2025), postoperative buttock claudication occurred in 26.1% (71/272) following EVAR. In the training set, multivariable logistic regression identified more than two distal internal iliac artery branches as an independent protective factor for postoperative buttock claudication (aOR, 0.15; 95% CI, 0.05–0.41), whereas peripheral arterial disease showed only a borderline association. Across ten machine learning algorithms, training/internal validation revealed complementary trade-offs (e.g., LightGBM highest F1 = 0.656; Random Forest accuracy = 0.822 but poor sensitivity; GBM sensitivity most closely balanced). The held-out test set then permitted optimal performance: the neural network (combined with simple logistic regression) achieved the best sensitivity (0.810) and near-top F1, CatBoost followed in accuracy and all round ‘net benefit’. Calibration was acceptable and decision-curve analysis provided clinically meaningful net benefit at various plausible thresholds. Model-agnostic explainability using SHAP for the neural network corroborated all of the above: embolization status and/or iliac involvement push risk upward, richer distal branch networks pull it down, and to the clinician at least, risk can be transparently and practically managed.

The risk architecture we observed is coherent with pelvic perfusion biology. Internal iliac artery (IIA) embolization compromises direct inflow to the superior and inferior gluteal arteries; thus, risk increases in a well-defined stepwise manner—none → unilateral → bilateral occlusion—precisely replicated by the SHAP dependence plots. Sacrificing both IIAs forces the gluteal musculature to rely on more circuitous collateral routes (profunda femoris–descending branch–gluteal anastomoses, obturator and lumbar being prime examples); these are frequently inadequate during exertion, leading to exertional gluteal ischemia [7,11]. A greater number of distal IIA branches (>2) likely represents a richer native collateral network; this reserve reduces the hemodynamic drop across the pelvis, and the strong protective association seen along with negative SHAP contributions is a direct reflection of it.

Aneurysm with iliac involvement plausibly signals more extensive aorto-iliac disease and all associated manipulation (landing zones, device coverage, adjunctive embolization), increasing the odds of such pelvic hypoperfusion [4,7]. Male sex may capture higher absolute muscular oxygen demand and various sex-linked vascular risk clustering, pushing the ischemic threshold higher during ambulation [14,15]. Hyperlipidemia aligns with microvascular dysfunction and impaired endothelial nitric-oxide pathway vasodilation, worsening any pre-existing supply–demand mismatch in the gluteal bed [16,17]. The borderline PAD signal is directionally consistent with a systemic atherosclerotic milieu—limited runoff/collateralization being part of that process [18,19]. Variables not retained as independent predictors (age, BMI, maximum aortic diameter, etc.) will always have or at least partially have weaker effects on the final clinical outcome of buttock claudication. Preserving at least one IIA, or any form of anatomically based preoperative planning, is a real and present clinical need.

Our findings align with—and extend—the existing EVAR literature on pelvic ischemia following internal iliac artery (IIA) interruption. Prior observational cohorts consistently report higher rates of buttock claudication associated with bilateral compared to unilateral IIA embolization; the protective role of some form of preserved pelvic inflow is frequently emphasized; our data reproduce this stepwise risk gradient, with a more richly branched distal IIA network serving as the specific mitigating factor [20,21]. The association between iliac-involved aneurysms and this claudication risk is concordant with reports of more complex aorto-iliac anatomy, longer ‘landing’ zones, and various forms of adjunctive embolization all increasing the potential for pelvic hypoperfusion [22]. Signals for a systemic vascular burden—hyperlipidemia being a prime example, borderline PAD if measured—were directionally consistent with work previously linking endothelial dysfunction and to a large extent limited collateral capacity to at least partially exercise-induced ischemia [23,24]. Variables such as age, BMI, and maximum aortic diameter have shown more heterogeneous effects; in our analysis they were all attenuated when true pelvic inflow/outflow anatomy and all related comorbidities were taken into account. Primary risk, it seems, is best defined by the anatomical or functional state of the pelvis itself.

Methodologically, our study adds several elements that are less commonly found in earlier reports The main contribution of the present study is not the discovery of entirely new risk factors, but the integration of clinically expected pelvic perfusion-related variables into an explainable prediction workflow and a research-use Shiny prototype for individualized risk estimation. First, we benchmarked ten supervised learning algorithms against multivariable logistic regression; complementary operating characteristics were demonstrated, with a specific neural network identified as the ‘best’ test set discriminator under locked thresholds [25,26]. Second, beyond simple discrimination, calibration and decision-curve analysis were emphasized; a clinically interpretable view of net benefit across all possible thresholds is what we sought to report—this very specific type of assessment being near constant in its absence from the literature [27,28]. Third, model-agnostic explainability was supplied; mechanistic plausibility (embolization status, iliac involvement increasing risk; more distal branches being protective) could be at least partially reconciled with the more abstract, individual level prediction logic of the model. Prior black-box applications lacked this form of explainability [29]. Finally, operationalizing the model into a Shiny tool bridges the near-perpetual gap between methodological promise and some form of bedside utility; preoperative planning and all subsequent patient counseling, based on transparent risk estimates, are both improved by it [30].

Across algorithms, operating characteristics were complementary rather than uniformly superior. On internal validation, LightGBM maximized F1 (0.656) with a near-perfect balance of sensitivity and specificity, Random Forest achieved the highest accuracy (0.822) by pushing towards some form of specificity (low false positives), and GBM served as the recall prioritizer (sensitivity 0.880). The held-out test set confirmed these trade-offs: CatBoost had the best accuracy and near-maximal specificity, but a moderate F1; Random Forest itself pushed specificity even higher, sensitivity dropping considerably (0.238, F1~0.323). Neural network delivered the highest sensitivity, the top F1 being attained under this screening-oriented objective. Calibration analyses were generally acceptable, decision-curve analysis showing positive net benefit for at least some clinically plausible thresholds; real world utility beyond simple discrimination was supported. Model selection followed a locked-threshold protocol to prevent test leakage; AUROC, F1 and calibration all factored into the final decision, with the neural network as the primary model chosen. Practically two operating regimes are defensible: preoperative screening/counseling type sensitivity, and more ‘specific’ follow-up based on resource or intervention risk. Bootstrap 95% CIs for AUROC (1000 resamples) provided the last necessary uncertainty bound. Finally, while the neural network led in all aspects of performance, we believe a fully interpretable model is still the end goal of clinical modeling.

Model-agnostic SHAP analyses bridged the gap between statistical performance and bedside decision-making [31,32]. Globally, both bar and beeswarm plots consistently identified IIA embolization status, iliac involvement, and some measure of the distal IIA branch count as the dominant drivers of risk; directions of effect were concordant with known physiology and multivariable odds ratios. Dependence plots captured more clinically intuitive gradients (none → unilateral → bilateral embolization increasing risk, >2 distal branches decreasing it if at all), case-level force and waterfall plots then decomposed individual predictions into near-actionable contributors. These various forms of explanation support shared decision-making: patients flagged as high risk can have specific recommendations made to them. Clinicians may (i) prioritize IIA preservation (anatomically if possible), (ii) move towards staged or selective embolization, branched/parallel iliac strategies, or any form of alternative ‘landing’ plan, and (iii) tailor postoperative surveillance and to a point rehabilitation. Operationally, our Shiny tool packages this entire workflow—routine preoperative variables are accepted, a personalization of risk is returned with a concise explanation panel, and all or part of the decision-making process can be guided by either screening or confirmation style use cases.

Strengths include a dual-center cohort spanning eight and a half years, prespecified training-set screening (*p* < 0.05), evaluation of ten algorithms all under a locked-threshold protocol, comprehensive assessment beyond simple discrimination—calibration and decision-curve analysis—SHAP transparency, and a deployable web calculator. Several limitations warrant some form of caution. The retrospective design risks residual confounding and various types of outcome misclassification; standardized definitions help but do not fully eliminate this [33,34]. Center-specific devices, embolization techniques, and near-perfectly controlled perioperative pathways limit true generalizability. Because both centers shared similar institutional practice patterns, including patient selection, EVAR technique, embolization strategy, device use, and documentation habits, the model may not generalize to hospitals with different procedural approaches or patient populations. Some potentially informative imaging features (quantitative pelvic collateral scores, IIA diameter/flow type metrics) were not collected, symptom severity and/or duration were not modeled. The sample size and number of outcome events were limited, particularly in the held-out test set; therefore, comparisons among ten machine learning algorithms should be interpreted as exploratory, because a small number of reclassified patients could materially affect sensitivity, specificity, F1 score, and AUROC. Although repeated cross-validation and bootstrap AUROC confidence intervals were used to reduce uncertainty, larger cohorts and external validation are required to assess model stability and generalizability. Finally, DCA assumes constant threshold utilities across settings, cost-effectiveness being the ultimate clinical endpoint we would like to see analyzed.

Clinically, the model encourages preoperative perfusion-aware planning: preserve at least one IIA whenever possible, appraise distal branch anatomy proactively, and reserve bilateral embolization for carefully selected scenarios with some form of mitigation strategy in place. The tool can inform counseling regarding expected risk, guide the intensity of follow-up, and flag patients for early rehabilitation or more specific perfusion assessments. However, the model should be interpreted as a risk-prediction tool rather than evidence that modifying any individual predictor or changing a procedural strategy will necessarily reduce postoperative buttock claudication. Research priorities include (i) prospective multicenter external validation; (ii) enriching the predictors with imaging-derived pelvic collateral metrics and various device/technique details; (iii) extending beyond simple binary outcomes to time-to-event and severity-based outcomes; (iv) explicit clinical or health-economic utilities to define ‘optimal’ operating points; and (v) impact trials testing whether model-guided preservation of IIA’s reduces buttock claudication, with all other ischemic complications being the secondary endpoint. Integration into the electronic health record with automated data ingestion and real-time, interpretable SHAP style explanations is the practical next step in achieving sustainable clinical adoption.

## 5. Conclusions

In this dual-center retrospective cohort study, we developed a preliminary explainable prediction model for postoperative buttock claudication after EVAR. The model showed promising internal performance and identified clinically plausible pelvic perfusion-related predictors, including internal iliac artery embolization, iliac involvement, and distal internal iliac branch anatomy. However, because the study was based on retrospective data and internal validation, external validation, prospective testing, and clinical impact evaluation are required before the model or Shiny calculator can be used for routine clinical decision-making.

## Figures and Tables

**Table 1:** Characteristics of included patients.

Demographic Characteristics	Desc	No Buttock Claudication (N = 201)	Buttock Claudication (N = 71)	*p*
Site_of_aneurysm	Abdominal_aortic_aneurysm_with_iliac_artery_aneurysm	53 (26.4%)	34 (47.9%)	0.001
	Abdominal_aortic_aneurysm_without_iliac_artery_aneurysm	148 (73.6%)	37 (52.1%)	
Gender	Female	67 (33.3%)	15 (21.1%)	0.076
	Male	134 (66.7%)	56 (78.9%)	
Peripheral_arterial_disease	No	131 (65.2%)	32 (45.1%)	0.005
	Yes	70 (34.8%)	39 (54.9%)	
Number_of_internal_iliac_arteries_embolized	Bilateral_embolization	14 (7%)	10 (14.1%)	<0.001
	None	80 (39.8%)	10 (14.1%)	
	Unilateral_embolism	107 (53.2%)	51 (71.8%)	
Chronic_obstructive_pulmonary_disease	No	187 (93%)	69 (97.2%)	0.325
	Yes	14 (7%)	2 (2.8%)	
Chronic_kidney_disease	No	159 (79.1%)	55 (77.5%)	0.903
	Yes	42 (20.9%)	16 (22.5%)	
Antiplatelet	No	63 (31.3%)	26 (36.6%)	0.504
	Yes	138 (68.7%)	45 (63.4%)	
Hyperlipidemia	No	68 (33.8%)	9 (12.7%)	0.001
	Yes	133 (66.2%)	62 (87.3%)	
History_of_previous_abdominal_and_pelvic_surgery	No	112 (55.7%)	50 (70.4%)	0.042
	Yes	89 (44.3%)	21 (29.6%)	
Cardiovascular_disease	No	126 (62.7%)	43 (60.6%)	0.861
	Yes	75 (37.3%)	28 (39.4%)	
Marital_status	Married	92 (45.8%)	32 (45.1%)	1
	Other	109 (54.2%)	39 (54.9%)	
Smoking	No	160 (79.6%)	58 (81.7%)	0.837
	Yes	41 (20.4%)	13 (18.3%)	
Drinking	No	128 (63.7%)	45 (63.4%)	1
	Yes	73 (36.3%)	26 (36.6%)	
Cerebrovascular_disease	No	106 (52.7%)	33 (46.5%)	0.442
	Yes	95 (47.3%)	38 (53.5%)	
Hypertension	No	103 (51.2%)	42 (59.2%)	0.312
	Yes	98 (48.8%)	29 (40.8%)	
Diabetes	No	136 (67.7%)	43 (60.6%)	0.348
	Yes	65 (32.3%)	28 (39.4%)	
Number_of_distal_internal_iliac_artery_branches	≤2	104 (51.7%)	63 (88.7%)	<0.001
	>2	97 (48.3%)	8 (11.3%)	
Age	Mean ± SD	65.7 ± 7.6	65.0 ± 8.3	0.484
BMI	Mean ± SD	30.0 ± 3.8	29.8 ± 4.5	0.725
Maximum_diameter_of_abdominal_aorta	Mean ± SD	5.8 ± 0.6	5.7 ± 0.5	0.094

**Table 2:** Training set univariate and multivariate logistic regression results.

Demographic Characteristics	Desc	No Buttock Claudication (N = 141)	Buttock Claudication (N = 50)	OR (Univariable)	OR (Multivariable)
Site_of_aneurysm	Abdominal_aortic_aneurysm_without_iliac_artery_aneurysm	107 (75.9%)	26 (52%)		
	Abdominal_aortic_aneurysm_with_iliac_artery_aneurysm	34 (24.1%)	24 (48%)	2.90 (1.48–5.71, *p* = 0.002)	4.04 (1.73–9.40, *p* = 0.001)
Gender	Female	45 (31.9%)	7 (14%)		
	Male	96 (68.1%)	43 (86%)	2.88 (1.20–6.90, *p* = 0.018)	3.26 (1.20–8.86, *p* = 0.021)
Peripheral_arterial_disease	No	94 (66.7%)	21 (42%)		
	Yes	47 (33.3%)	29 (58%)	2.76 (1.42–5.35, *p* = 0.003)	2.19 (0.98–4.89, *p* = 0.055)
Number_of_internal_iliac_arteries_embolized	None	58 (41.1%)	7 (14%)		
	Unilateral_embolism	76 (53.9%)	36 (72%)	3.92 (1.63–9.45, *p* = 0.002)	3.86 (1.38–10.80, *p* = 0.010)
	Bilateral_embolization	7 (5%)	7 (14%)	8.29 (2.24–30.67, *p* = 0.002)	8.61 (1.78–41.68, *p* = 0.007)
Chronic_obstructive_pulmonary_disease	No	131 (92.9%)	48 (96%)		
	Yes	10 (7.1%)	2 (4%)	0.55 (0.12–2.58, *p* = 0.445)	
Chronic_kidney_disease	No	111 (78.7%)	41 (82%)		
	Yes	30 (21.3%)	9 (18%)	0.81 (0.36–1.86, *p* = 0.622)	
Antiplatelet	No	47 (33.3%)	22 (44%)		
	Yes	94 (66.7%)	28 (56%)	0.64 (0.33–1.23, *p* = 0.179)	
Hyperlipidemia	No	50 (35.5%)	5 (10%)		
	Yes	91 (64.5%)	45 (90%)	4.95 (1.84–13.26, *p* = 0.002)	5.66 (1.84–17.37, *p* = 0.002)
History_of_previous_abdominal_and_pelvic_surgery	No	77 (54.6%)	33 (66%)		
	Yes	64 (45.4%)	17 (34%)	0.62 (0.32–1.21, *p* = 0.163)	
Cardiovascular_disease	No	87 (61.7%)	27 (54%)		
	Yes	54 (38.3%)	23 (46%)	1.37 (0.72–2.63, *p* = 0.341)	
Marital_status	other	81 (57.4%)	27 (54%)		
	married	60 (42.6%)	23 (46%)	1.15 (0.60–2.20, *p* = 0.673)	
Smoking	No	112 (79.4%)	42 (84%)		
	Yes	29 (20.6%)	8 (16%)	0.74 (0.31–1.74, *p* = 0.484)	
Drinking	No	95 (67.4%)	33 (66%)		
	Yes	46 (32.6%)	17 (34%)	1.06 (0.54–2.11, *p* = 0.859)	
Cerebrovascular_disease	No	82 (58.2%)	25 (50%)		
	Yes	59 (41.8%)	25 (50%)	1.39 (0.73–2.66, *p* = 0.319)	
Hypertension	No	78 (55.3%)	30 (60%)		
	Yes	63 (44.7%)	20 (40%)	0.83 (0.43–1.59, *p* = 0.566)	
Diabetes	No	94 (66.7%)	31 (62%)		
	Yes	47 (33.3%)	19 (38%)	1.23 (0.63–2.40, *p* = 0.551)	
Number_of_distal_internal_iliac_artery_branches	≤2	72 (51.1%)	43 (86%)		
	>2	69 (48.9%)	7 (14%)	0.17 (0.07–0.40, *p* < 0.001)	0.15 (0.05–0.41, *p* < 0.001)
Age	Mean ± SD	66.1 ± 7.1	64.4 ± 8.9	0.97 (0.93–1.01, *p* = 0.162)	
BMI	Mean ± SD	30.0 ± 3.8	30.3 ± 4.0	1.02 (0.94–1.12, *p* = 0.596)	
Maximum_diameter_of_abdominal_aorta	Mean ± SD	5.8 ± 0.6	5.7 ± 0.5	0.82 (0.46–1.46, *p* = 0.501)	

**Table 3:** Training set evaluation metrics.

Model	Threshold	Accuracy	Sensitivity	Specificity	Precision	F1
Logistic	0.255179063336043	0.754	0.8	0.738	0.519	0.63
SVM	0.383768854671191	0.759	0.8	0.745	0.526	0.635
GBM	0.176710321609796	0.723	0.88	0.667	0.484	0.624
NeuralNetwork	0.283961858247819	0.754	0.8	0.738	0.519	0.63
RandomForest	0.5	0.822	0.42	0.965	0.808	0.553
Xgboost	0.228472709655762	0.733	0.86	0.688	0.494	0.628
KNN	0.368037682277079	0.78	0.76	0.787	0.559	0.644
AdaBoost	0.378156889570457	0.728	0.86	0.681	0.489	0.623
LightGBM	0.268290810466596	0.764	0.86	0.73	0.531	0.656
CatBoost	0.564815031821062	0.691	0.84	0.638	0.452	0.587

**Table 4:** Test set evaluation metrics.

Model	Threshold	Accuracy	Sensitivity	Specificity	Precision	F1
Logistic	0.217765116303309	0.667	0.81	0.617	0.425	0.557
SVM	0.285017690753319	0.704	0.714	0.7	0.455	0.556
GBM	0.250373966962187	0.704	0.714	0.7	0.455	0.556
NeuralNetwork	0.255776618280099	0.667	0.81	0.617	0.425	0.557
RandomForest	0.5	0.741	0.238	0.917	0.5	0.323
Xgboost	0.236851990222931	0.704	0.714	0.7	0.455	0.556
KNN	0.284118041139883	0.691	0.619	0.717	0.433	0.51
AdaBoost	0.412347543018369	0.691	0.714	0.683	0.441	0.545
LightGBM	0.227497064822974	0.642	0.667	0.633	0.389	0.491
CatBoost	0.568341230397805	0.79	0.476	0.9	0.625	0.541

## Data Availability

The data analyzed and the codes used during the current study are available from the corresponding author on reasonable request.

## Abbreviations

The following abbreviations are used in this manuscript:

AAA	abdominal aortic aneurysm
BMI	body mass index
CI	confidence interval
DCA	decision-curve analysis
EVAR	endovascular aneurysm repair
ML	machine learning
ROC	receiver operating characteristic
OR	odds ratio
SHAP	SHapley Additive exPlanations
PAD	peripheral arterial disease
